# Crystal structure, DFT study and Hirshfeld surface analysis of ethyl 6-chloro-2-eth­oxy­quinoline-4-carboxyl­ate

**DOI:** 10.1107/S2056989019007473

**Published:** 2019-05-31

**Authors:** Younos Bouzian, Khalid Karrouchi, El Hassane Anouar, Rachid Bouhfid, Suhana Arshad, El Mokhtar Essassi

**Affiliations:** a Laboratory of Heterocyclic Organic Chemistry, URAC 21, Pole of Competence, Pharmacochemistry, Av Ibn Battouta, BP 1014, Faculty of Sciences, Mohammed V University, Rabat, Morocco; b Laboratory of Plant Chemistry, Organic and Bioorganic Synthesis, URAC23, Faculty of Science, BP 1014, GEOPAC Research Center, Mohammed V University, Rabat, Morocco; c Department of Chemistry, College of Science and Humanities, Prince Sattam Bin Abdulaziz University, PO Box 830, Al Kharj, Saudi Arabia; d Moroccan Foundation for Advanced Science, Innovation and Research (MASCIR), Rabat, Morocco; e X-ray Crystallography Unit, School of Physics, Universiti Sains Malaysia, 11800 USM, Penang, Malaysia

**Keywords:** crystal structure, quinoline, offset π–π inter­actions, Hirshfeld surface analysis, DFT

## Abstract

In the title quinoline derivative, C_14_H_14_ClNO_3_, there is an intra­molecular C—H⋯O hydrogen bond forming an *S*(6) graph-set motif. The mol­ecule is essentially planar with the mean plane of the ethyl acetate group making a dihedral angle of 5.02 (3)° with the ethyl 6-chloro-2-eth­oxy­quinoline mean plane. In the crystal, offset π–π inter­actions with a centroid-to-centroid distance of 3.4731 (14) Å link inversion-related mol­ecules into columns along the *c*-axis direction. Hirshfeld surface analysis indicates that H⋯H contacts make the largest contribution (50.8%) to the Hirshfeld surface.

## Abstract 2

The title quinoline derivative is essentially planar with the ethyl acetate mean plane making a dihedral angle of 5.02 (3)° with the ethyl 6-chloro-2-eth­oxy­quinoline mean plane. In the crystal, offset π–π inter­actions involving inversion-related pyridine rings [centroid-to-centroid distance = 3.4731 (14) Å] link the mol­ecules into columns along the *c*-axis direction.

## Chemical context   

Quinoline derivatives represent an important class of bioactive heterocyclic compounds in the field of pharmaceuticals (Chu *et al.*, 2019 ▸). Quinoline derivatives possess various pharmacological properties such as anti­bacterial (Panda *et al.*, 2015 ▸), anti-HCV (Cannalire *et al.*, 2016 ▸), anti­viral (Sekgota *et al.*, 2017 ▸), anti­cancer (Tang *et al.*, 2018 ▸), anti­malarial (van Heerden *et al.*, 2012 ▸), anti­leishmanial (Palit *et al.*, 2009 ▸), anti­tubecular (Xu *et al.*, 2017 ▸), anti-inflammatory (de Santos *et al.*, 2015 ▸) and anti-Alzheimer’s (Bolognesi *et al.*, 2007 ▸) activities. The present work is a continuation of our research work devoted to the synthesis and crystal structure of heterocyclic derivatives (Bouzian *et al.*, 2018 ▸; Chkirate *et al.* 2019*a* ▸,*b* ▸). As part of our studies in this area, we prepared the title compound by reacting ethyl 6-chloro-2-oxo-1,2-di­hydro­quinoline-4-carboxyl­ate with bromo­ethane in the presence of a catalytic qu­antity of tetra-*n*-butyl­ammonium bromide. We report herein on its crystal and mol­ecular structures along with the Hirshfeld surface analysis.
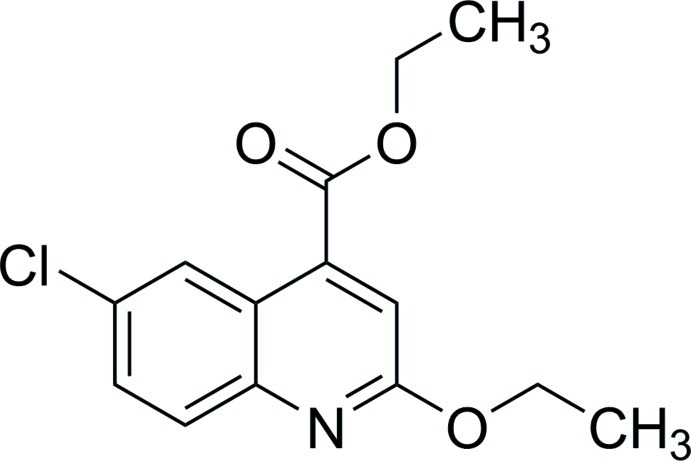


## Structural commentary   

**Figure 1:**
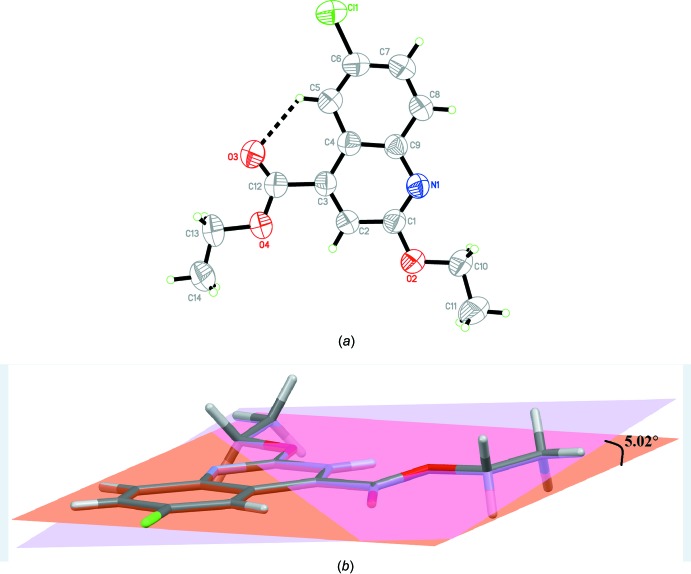
(*a*) The mol­ecular structure of the title compound, with the atom labelling and displacement ellipsoids drawn at the 50% probability level. The dashed line represents the intra­molecular C—H⋯O inter­action (Table 1 ▸). (*b*) The essentially planar structure of the title compound.

The mol­ecular structure of the title compound is shown in Fig. 1 ▸ *a*. The mol­ecule consists of a quinoline fused-ring system (N1/C1–C9) with meth­oxy­ethane (O2/C10/C11), ethyl acetate (O3/O4/C13/C14) and a chlorine atom (Cl1) substituents. The intra­molecular C5—H5*A*⋯O3 hydrogen bond (Table 1 ▸) forms an *S*(6) graph-set motif, stabilizing the mol­ecular structure and preventing free rotation between the 6-chloro­quinoline ring (Cl1/N1/C1–C9) and the ethyl acetate (O3/O4/C12–C14) moiety. Additionally, the presence of this intra­molecular C—H⋯O inter­action leads to an essentially planar mol­ecular structure (Fig. 1 ▸ *b*), where the ethyl acetate (O3/O4/C12–C14) mean plane is twisted slightly at a dihedral angle of 5.02 (3)° with respect to the mean plane of the ethyl 6-chloro-2-eth­oxy­quinoline (Cl1/O2/C1–C11) moiety. This essentially planar mol­ecular structure may be considered an important binding mode that can enhance biological activity (Bierbach *et al.*, 1999 ▸).

## Supra­molecular features   

**Figure 2:**
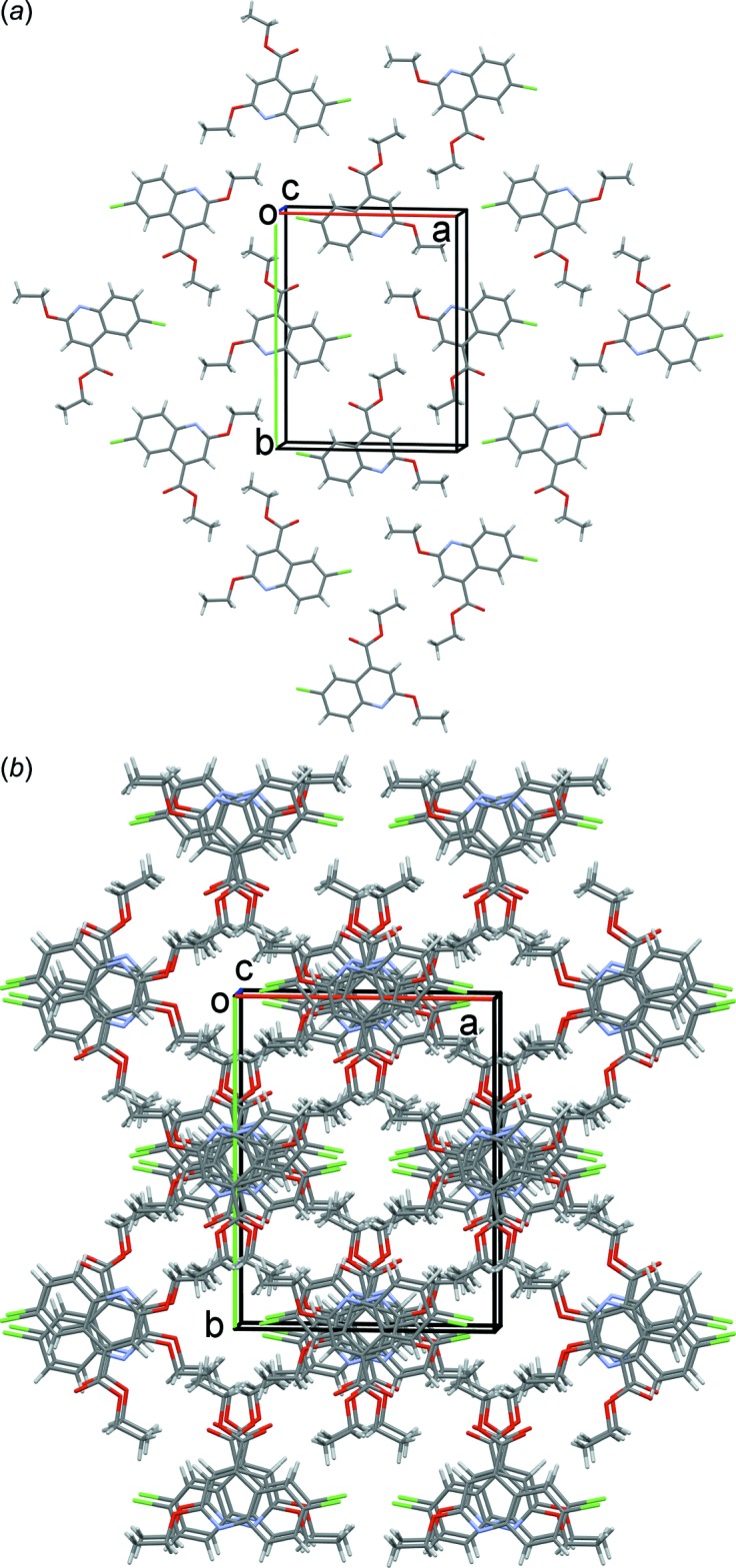
(*a*) A partial view along the *c* axis of the crystal packing of the title compound. (*b*) A view along the *c* axis of the crystal packing of the title compound.

In the crystal, mol­ecules lie in a plane parallel to the (10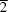) crystallographic plane (Fig. 2 ▸ *a*). They are linked by offset π–π inter­actions (Fig. 2 ▸ *b*) involving inversion-related pyridine rings. These inter­actions link the mol­ecules into columns up the *c*-axis direction with a centroid-to-centroid (*Cg*⋯*Cg* ^i^) distance of 3.4731 (14) Å [*Cg* is centroid of the N1/C1–C4/C9 ring, inter­planar distance = 3.397 (1) Å, offset = 0.722 Å; symmetry code (i): −x + 1, −y, −z + 1].

## Hirshfeld surface analysis   

**Figure 3:**
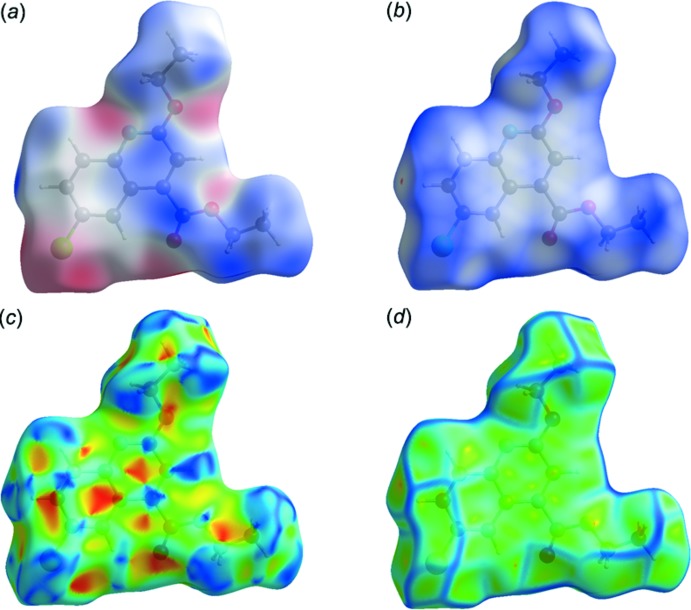
Hirshfeld surface of the title compound mapped over: (*a*) electrostatic potential, *(b) d*_norm_, *(c)* shape-index and (*d*) curvedness.

The Hirshfeld surface analysis (Spackman & Jayatilaka, 2009 ▸) and the associated two-dimensional fingerprint plots (McKinnon *et al.*, 2007 ▸) were performed with *CrystalExplorer17* (Turner *et al.*, 2017 ▸). Inter­nal and external (*d*_i_ and *d*_e_) contact distances from the Hirshfeld surface to the nearest atom inside and outside enables the analysis of the inter­molecular inter­actions through the mapping of *d*_norm_. The Hirshfeld surfaces (HS) mapped over the electrostatic potential (−0.0534 to 0.0319 atomic units) and *d*_norm_ (−0.0210 to 1.4779 arbitrary units) are shown in Fig. 3 ▸ *a* and 3*b*. The red spots on the Hirshfeld surface indicate inter­actions involved in H⋯O contacts. The π–π stacking is confirmed by the small blue regions surrounding bright red spots in the aromatic ring in Fig. 3 ▸ *c*, the Hirshfeld surface mapped over the shape-index, and by the flat regions around the aromatic regions in Fig. 3 ▸ *d*, the Hirshfeld surface mapped over the curvedness.

**Figure 4:**
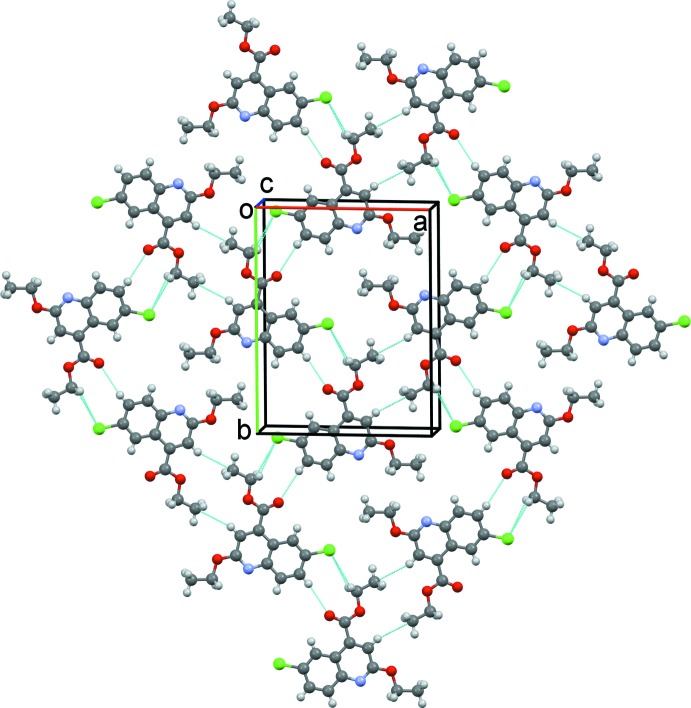
A view of the inter­molecular contacts (dashed lines) in the crystal of the title compound. They are all longer by 0.02 Å than the sum of the van der Waals radii of the individual atoms.

**Figure 5:**
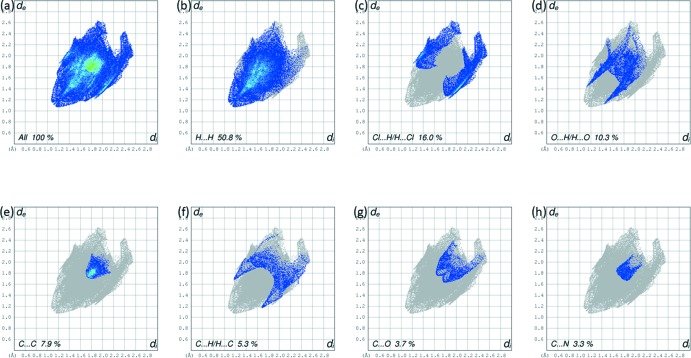
(*a*) The two-dimensional fingerprint plot of the title compound, and the fingerprint plots delineated into: (*b*) H⋯H (50.8%), (*c*) Cl⋯H/H⋯Cl (16.0%), (*d*) O⋯H/H⋯O (10.3%), (*e*) C⋯C (7.9%), (*f*) C⋯H/H⋯C (5.3%), (*g*) C⋯O (3.7%) and (*h*) C⋯N (3.3%) contacts.

There are no significant classical inter­molecular contacts present in the crystal according to the analysis of the crystal structure using *PLATON* (Spek, 2009 ▸). However, from the Hirshfeld surface analysis and the two-dimensional fingerprint plots it can be seen that H⋯H, C⋯H, Cl⋯H and O⋯H contacts (Fig. 4 ▸) contribute to the cohesion of the crystal structure. The two-dimensional fingerprint plots are given in Fig. 5 ▸. The two-dimensional fingerprint of the (*d_i_*, *d_e_*) points associated with the hydrogen atoms is shown in Fig. 5 ▸ *b*. It is characterized by an end point that points to the origin, indicating the presence of the H⋯H contacts that contribution 50.8%. The Cl⋯H/H⋯Cl contacts between the chlorine atoms inside the Hirshfeld surface and the hydrogen atoms outside the surface and *vice versa* contribute 16.0% (Fig. 5 ▸ *c*). The O⋯H/H⋯O (10.3%) plot shows two symmetrical wings on the left and right sides (Fig. 5 ▸ *d*). The C⋯C contacts contribute 7.9% (Fig. 5 ▸ *e*), the C⋯H/H⋯C contacts contribute 5.3% (Fig. 5 ▸ *e*), followed by the C⋯O contacts at 3.7% (Fig. 5 ▸ *g*) and the C⋯N contacts at 3.3% (Fig. 3 ▸ *h*).

## DFT study   

**Figure 6:**
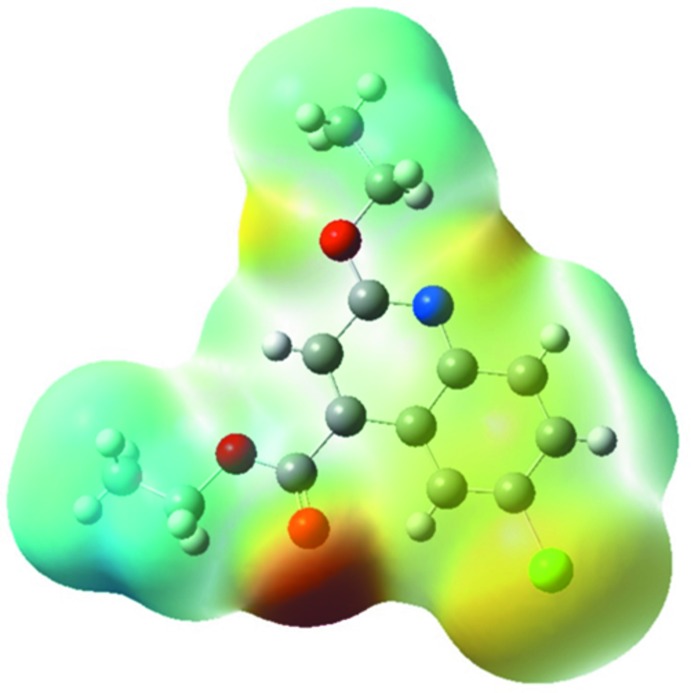
EPS of the title compound obtained at the B3LYP/6–31+G(d,p) level of theory.

The electrostatic potential surface (ESP) was also calculated using DFT methods at the B3LYP/6-311+G(d,p) level of theory using the *Gaussian 09* package (Frisch *et al.*, 2009 ▸). The negative region on the electrostatic potential appears in red and corresponds to hydrogen-bond acceptors, while the positive region of electrostatic potential appears in blue and corresponds to hydrogen-bond donors (Fig. 6 ▸).

## Database survey   

A search of the Cambridge Structural Database (CSD, Version 5.40, last update May 2019; Groom *et al.*, 2016 ▸) for the 6-chloro­quinoline skeleton gave 100 hits, including 6-chloro­quinoline itself (CSD refcode CLQUIN; Merlino, 1968 ▸). Only a limited number of these structures are similar to the title compound. There are no compounds with a 6-chloro-2-eth­oxy­quinoline moiety and only four compounds with a 6-chloro-2-meth­oxy­quinoline moiety. These include, 1-{6-chloro-2-[(2-chloro-8-methyl­quinolin-3-yl)meth­oxy]-4-phen­yl­quinolin-3-yl}ethanone (DUVJEK; Khan *et al.*, 2010*a* ▸), ethyl 6-chloro-2-[(2-chloro-7,8-di­methyl­quinolin-3-yl)meth­oxy]-4-phenyl­quinoline-3-carboxyl­ate (KUVFEN; Khan *et al.*, 2010*b* ▸), 1-{6-chloro-2-[(2-chloro­quinolin-3-yl)meth­oxy]-4-phenyl­quinolin-3-yl}ethanone (YUQTAG; Khan *et al.*, 2010*c* ▸), and 1-{6-chloro-2-[(2-chloro-6-methyl­quinolin-3-yl)meth­oxy]-4-phenyl­quinolin-3-yl}ethanone (YUQVIQ; Khan *et al.*, 2010*d* ▸). Two other relevant compounds with an ethyl carboxyl­ate substituent include ethyl 2,6-di­chloro-4-phenyl­quinoline-3-carboxyl­ate (DUKKUQ; Roopan *et al.*, 2009 ▸) and ethyl 6-chloro-2-methyl-4-phenyl­quinoline-3-carboxyl­ate (DUKJEZ; Subashini *et al.*, 2009 ▸). In the crystals of all of the above mentioned compounds, mol­ecules are linked by offset π–π inter­actions involving inversion-related quinoline units.

## Synthesis and crystallization   

A solution of 0.5 g (1.99 mmol) of ethyl 6-chloro-2-oxo-1,2-di­hydro­quinoline-4-carboxyl­ate in 25 ml of DMF was mixed with 0.3 ml (3.98 mmol) of bromo­ethane, 0.55 g (3.98 mmol) of K_2_CO_3_ and 0.06 g (0.199 mmol) of tetra-*n*-butyl­ammonium bromide (TBAB). The reaction mixture was stirred at room temperature in DMF for 24 h. After removal of salts by filtration, the DMF was evaporated under reduced pressure and the residue obtained was dissolved in di­chloro­methane·The organic phase was dried over Na_2_SO_4_ then concentrated *in vacuo*. The resulting mixture was chromatographed on a silica gel column [eluent: ethyl acetate/hexane (1:9 *v*/*v*)]. Colourless crystals were obtained when the solvent was allowed to evaporate (yield: 32%).

## Refinement   

Crystal data, data collection and structure refinement details are summarized in Table 2 ▸. All H atoms were positioned geometrically and refined using a riding model: C—H = 0.93-0.97 Å with *U*_iso_(H) = 1.5*U*_eq_(C-meth­yl) and 1.2*U*_eq_(C) for other H atoms. A rotating group model was applied to the methyl groups.

## Supplementary Material

Crystal structure: contains datablock(s) I. DOI: 10.1107/S2056989019007473/mw2143sup1.cif

Structure factors: contains datablock(s) I. DOI: 10.1107/S2056989019007473/mw2143Isup2.hkl

Supporting information file. DOI: 10.1107/S2056989019007473/mw2143Isup3.cml

CCDC reference: 1890687

Additional supporting information:  crystallographic information; 3D view; checkCIF report

## Figures and Tables

**Table 1:** Hydrogen-bond geometry (Å, °)

*D*—H⋯*A*	*D*—H	H⋯*A*	*D*⋯*A*	*D*—H⋯*A*
C5—H5*A*⋯O3	0.93	2.24	2.872 (4)	125

**Table 2:** Experimental details

Crystal data	
Chemical formula	C_14_H_14_ClNO_3_
*M*_r_	279.71
Crystal system, space group	Monoclinic, *C*2/*c*
Temperature (K)	296
*a*, *b*, *c* (Å)	14.2634 (7), 16.0124 (7), 13.7732 (6)
β (°)	117.748 (2)
*V* (Å^3^)	2783.9 (2)
*Z*	8
Radiation type	Mo *K*α
μ (mm^−1^)	0.28
Crystal size (mm)	0.50 × 0.47 × 0.37

Data collection	
Diffractometer	Bruker SMART APEXII DUO CCD area-detector
Absorption correction	Multi-scan (*SADABS*; Bruker, 2009 ▸)
No. of measured, independent and observed [*I* > 2σ(*I*)] reflections	45458, 3190, 2228
*R*_int_	0.029
(sin θ/λ)_max_ (Å^−1^)	0.650

Refinement	
*R*[*F* ^2^ > 2σ(*F* ^2^)], *wR*(*F* ^2^), *S*	0.058, 0.203, 1.10
No. of reflections	3190
No. of parameters	174
H-atom treatment	H-atom parameters constrained
Δρ_max_, Δρ_min_ (e Å^−3^)	0.30, −0.32

Computer programs: *APEX2* and *SAINT* (Bruker, 2009 ▸), *SHELXS* and *SHELXTL* (Sheldrick, 2008 ▸), *SHELXL2014* (Sheldrick, 2015 ▸), *Mercury* (Macrae *et al.*, 2008 ▸) and *PLATON* (Spek, 2009 ▸).

## supplementary crystallographic information

### Crystal data

**Table:**

C_14_H_14_ClNO_3_	*F*(000) = 1168
*M**_r_* = 279.71	*D*_x_ = 1.335 Mg m^−^^3^
Monoclinic, *C*2/*c*	Mo *K*α radiation, λ = 0.71073 Å
*a* = 14.2634 (7) Å	Cell parameters from 9928 reflections
*b* = 16.0124 (7) Å	θ = 2.5–24.5°
*c* = 13.7732 (6) Å	µ = 0.28 mm^−^^1^
β = 117.748 (2)°	*T* = 296 K
*V* = 2783.9 (2) Å^3^	Block, colourless
*Z* = 8	0.50 × 0.47 × 0.37 mm

### Data collection

**Table:**

Bruker SMART APEXII DUO CCD area-detector diffractometer	2228 reflections with *I* > 2σ(*I*)
Radiation source: fine-focus sealed tube	*R*_int_ = 0.029
φ and ω scans	θ_max_ = 27.5°, θ_min_ = 2.1°
Absorption correction: multi-scan (*SADABS*; Bruker, 2009)	*h* = −18→18
	*k* = −20→20
45458 measured reflections	*l* = −17→17
3190 independent reflections	

### Refinement

**Table:**

Refinement on *F*^2^	Primary atom site location: structure-invariant direct methods
Least-squares matrix: full	Secondary atom site location: difference Fourier map
*R*[*F*^2^ > 2σ(*F*^2^)] = 0.058	Hydrogen site location: inferred from neighbouring sites
*wR*(*F*^2^) = 0.203	H-atom parameters constrained
*S* = 1.10	*w* = 1/[σ^2^(*F*_o_^2^) + (0.0854*P*)^2^ + 2.5795*P*] where *P* = (*F*_o_^2^ + 2*F*_c_^2^)/3
3190 reflections	(Δ/σ)_max_ < 0.001
174 parameters	Δρ_max_ = 0.30 e Å^−^^3^
0 restraints	Δρ_min_ = −0.32 e Å^−^^3^

### Special details

**Table:**

Geometry. All esds (except the esd in the dihedral angle between two l.s. planes) are estimated using the full covariance matrix. The cell esds are taken into account individually in the estimation of esds in distances, angles and torsion angles; correlations between esds in cell parameters are only used when they are defined by crystal symmetry. An approximate (isotropic) treatment of cell esds is used for estimating esds involving l.s. planes.

### Fractional atomic coordinates and isotropic or equivalent isotropic displacement parameters (Å^2^)

**Table:**

	*x*	*y*	*z*	*U*_iso_*/*U*_eq_	
N1	0.56133 (16)	0.10176 (12)	0.41064 (15)	0.0584 (5)	
Cl1	0.10792 (6)	0.02217 (6)	0.16402 (10)	0.1199 (5)	
O2	0.73447 (14)	0.05835 (11)	0.51005 (15)	0.0707 (5)	
O3	0.39144 (18)	−0.18904 (14)	0.3085 (2)	0.1148 (9)	
O4	0.55981 (16)	−0.20506 (11)	0.42458 (19)	0.0872 (6)	
C1	0.63047 (19)	0.04194 (15)	0.44989 (19)	0.0572 (6)	
C2	0.60502 (19)	−0.04443 (15)	0.43555 (19)	0.0577 (5)	
H2A	0.6582	−0.0845	0.4655	0.069*	
C3	0.50216 (18)	−0.06779 (14)	0.37760 (18)	0.0547 (5)	
C4	0.42215 (18)	−0.00513 (14)	0.33217 (17)	0.0542 (5)	
C5	0.3122 (2)	−0.02093 (17)	0.2719 (2)	0.0645 (6)	
H5A	0.2876	−0.0756	0.2569	0.077*	
C6	0.2427 (2)	0.04352 (19)	0.2358 (2)	0.0741 (7)	
C7	0.2757 (2)	0.12657 (19)	0.2548 (2)	0.0759 (7)	
H7A	0.2264	0.1697	0.2294	0.091*	
C8	0.3812 (2)	0.14362 (16)	0.3112 (2)	0.0682 (7)	
H8A	0.4037	0.1989	0.3233	0.082*	
C9	0.45660 (19)	0.07928 (14)	0.35138 (18)	0.0554 (5)	
C10	0.7649 (2)	0.14510 (18)	0.5310 (2)	0.0761 (8)	
H10A	0.7270	0.1724	0.5651	0.091*	
H10B	0.7486	0.1737	0.4629	0.091*	
C11	0.8808 (3)	0.1471 (2)	0.6056 (4)	0.1162 (14)	
H11A	0.9045	0.2041	0.6195	0.174*	
H11B	0.9172	0.1183	0.5720	0.174*	
H11C	0.8957	0.1203	0.6736	0.174*	
C12	0.4759 (2)	−0.15957 (16)	0.3647 (2)	0.0649 (6)	
C13	0.5438 (3)	−0.29493 (18)	0.4194 (4)	0.1060 (12)	
H13A	0.4951	−0.3092	0.4477	0.127*	
H13B	0.5133	−0.3135	0.3437	0.127*	
C14	0.6421 (3)	−0.3350 (2)	0.4822 (6)	0.181 (3)	
H14A	0.6739	−0.3139	0.5559	0.271*	
H14B	0.6882	−0.3243	0.4503	0.271*	
H14C	0.6311	−0.3941	0.4830	0.271*	

### Atomic displacement parameters (Å^2^)

**Table:**

	*U*^11^	*U*^22^	*U*^33^	*U*^12^	*U*^13^	*U*^23^
N1	0.0598 (11)	0.0493 (10)	0.0607 (11)	−0.0024 (8)	0.0234 (9)	0.0043 (8)
Cl1	0.0539 (4)	0.0945 (7)	0.1717 (10)	0.0034 (4)	0.0193 (5)	0.0156 (6)
O2	0.0549 (10)	0.0585 (10)	0.0825 (11)	−0.0052 (8)	0.0183 (9)	0.0045 (8)
O3	0.0763 (14)	0.0587 (12)	0.152 (2)	−0.0105 (10)	0.0054 (14)	−0.0116 (13)
O4	0.0709 (12)	0.0473 (10)	0.1220 (16)	0.0002 (8)	0.0268 (11)	0.0063 (10)
C1	0.0556 (13)	0.0541 (12)	0.0576 (12)	−0.0060 (10)	0.0227 (10)	0.0019 (10)
C2	0.0561 (13)	0.0517 (12)	0.0624 (13)	0.0011 (10)	0.0251 (11)	0.0036 (10)
C3	0.0592 (13)	0.0486 (12)	0.0561 (12)	−0.0037 (10)	0.0267 (10)	−0.0002 (9)
C4	0.0579 (13)	0.0540 (12)	0.0509 (11)	−0.0003 (10)	0.0255 (10)	0.0019 (9)
C5	0.0572 (14)	0.0617 (14)	0.0696 (15)	−0.0041 (11)	0.0253 (12)	−0.0001 (11)
C6	0.0533 (14)	0.0739 (17)	0.0837 (18)	0.0037 (12)	0.0223 (13)	0.0067 (14)
C7	0.0659 (16)	0.0661 (16)	0.0890 (19)	0.0116 (13)	0.0303 (14)	0.0087 (14)
C8	0.0661 (15)	0.0539 (13)	0.0782 (16)	0.0041 (11)	0.0283 (13)	0.0067 (12)
C9	0.0586 (13)	0.0519 (12)	0.0543 (12)	−0.0009 (10)	0.0251 (10)	0.0032 (9)
C10	0.0634 (15)	0.0614 (15)	0.0877 (18)	−0.0145 (12)	0.0218 (14)	−0.0014 (13)
C11	0.068 (2)	0.097 (3)	0.140 (3)	−0.0215 (18)	0.012 (2)	0.009 (2)
C12	0.0628 (15)	0.0534 (13)	0.0731 (15)	−0.0026 (11)	0.0272 (12)	−0.0030 (11)
C13	0.100 (2)	0.0447 (15)	0.152 (3)	−0.0038 (15)	0.041 (2)	0.0042 (17)
C14	0.084 (3)	0.057 (2)	0.328 (8)	0.0106 (18)	0.034 (4)	0.033 (3)

### Geometric parameters (Å, º)

**Table:**

N1—C1	1.298 (3)	C6—C7	1.394 (4)
N1—C9	1.375 (3)	C7—C8	1.361 (4)
Cl1—C6	1.737 (3)	C7—H7A	0.9300
O2—C1	1.346 (3)	C8—C9	1.404 (3)
O2—C10	1.444 (3)	C8—H8A	0.9300
O3—C12	1.186 (3)	C10—C11	1.486 (4)
O4—C12	1.313 (3)	C10—H10A	0.9700
O4—C13	1.454 (3)	C10—H10B	0.9700
C1—C2	1.420 (3)	C11—H11A	0.9600
C2—C3	1.356 (3)	C11—H11B	0.9600
C2—H2A	0.9300	C11—H11C	0.9600
C3—C4	1.427 (3)	C13—C14	1.413 (5)
C3—C12	1.506 (3)	C13—H13A	0.9700
C4—C5	1.415 (3)	C13—H13B	0.9700
C4—C9	1.420 (3)	C14—H14A	0.9600
C5—C6	1.355 (4)	C14—H14B	0.9600
C5—H5A	0.9300	C14—H14C	0.9600
			
C1—N1—C9	117.3 (2)	C8—C9—C4	119.3 (2)
C1—O2—C10	117.0 (2)	O2—C10—C11	107.1 (2)
C12—O4—C13	116.2 (2)	O2—C10—H10A	110.3
N1—C1—O2	121.2 (2)	C11—C10—H10A	110.3
N1—C1—C2	124.4 (2)	O2—C10—H10B	110.3
O2—C1—C2	114.4 (2)	C11—C10—H10B	110.3
C3—C2—C1	119.1 (2)	H10A—C10—H10B	108.6
C3—C2—H2A	120.4	C10—C11—H11A	109.5
C1—C2—H2A	120.4	C10—C11—H11B	109.5
C2—C3—C4	119.3 (2)	H11A—C11—H11B	109.5
C2—C3—C12	118.7 (2)	C10—C11—H11C	109.5
C4—C3—C12	122.0 (2)	H11A—C11—H11C	109.5
C5—C4—C9	118.2 (2)	H11B—C11—H11C	109.5
C5—C4—C3	125.0 (2)	O3—C12—O4	122.8 (3)
C9—C4—C3	116.8 (2)	O3—C12—C3	125.9 (3)
C6—C5—C4	120.1 (2)	O4—C12—C3	111.3 (2)
C6—C5—H5A	120.0	C14—C13—O4	109.3 (3)
C4—C5—H5A	120.0	C14—C13—H13A	109.8
C5—C6—C7	122.1 (3)	O4—C13—H13A	109.8
C5—C6—Cl1	119.0 (2)	C14—C13—H13B	109.8
C7—C6—Cl1	118.8 (2)	O4—C13—H13B	109.8
C8—C7—C6	119.0 (3)	H13A—C13—H13B	108.3
C8—C7—H7A	120.5	C13—C14—H14A	109.5
C6—C7—H7A	120.5	C13—C14—H14B	109.5
C7—C8—C9	121.2 (3)	H14A—C14—H14B	109.5
C7—C8—H8A	119.4	C13—C14—H14C	109.5
C9—C8—H8A	119.4	H14A—C14—H14C	109.5
N1—C9—C8	117.6 (2)	H14B—C14—H14C	109.5
N1—C9—C4	123.1 (2)		
			
C9—N1—C1—O2	−179.1 (2)	C6—C7—C8—C9	−0.8 (4)
C9—N1—C1—C2	0.1 (3)	C1—N1—C9—C8	178.3 (2)
C10—O2—C1—N1	1.5 (3)	C1—N1—C9—C4	0.1 (3)
C10—O2—C1—C2	−177.7 (2)	C7—C8—C9—N1	−177.9 (2)
N1—C1—C2—C3	−0.2 (4)	C7—C8—C9—C4	0.4 (4)
O2—C1—C2—C3	179.0 (2)	C5—C4—C9—N1	178.8 (2)
C1—C2—C3—C4	0.2 (3)	C3—C4—C9—N1	−0.1 (3)
C1—C2—C3—C12	−178.9 (2)	C5—C4—C9—C8	0.6 (3)
C2—C3—C4—C5	−178.9 (2)	C3—C4—C9—C8	−178.3 (2)
C12—C3—C4—C5	0.2 (4)	C1—O2—C10—C11	175.5 (3)
C2—C3—C4—C9	−0.1 (3)	C13—O4—C12—O3	−0.4 (5)
C12—C3—C4—C9	179.0 (2)	C13—O4—C12—C3	179.5 (3)
C9—C4—C5—C6	−1.2 (4)	C2—C3—C12—O3	−172.8 (3)
C3—C4—C5—C6	177.5 (2)	C4—C3—C12—O3	8.1 (4)
C4—C5—C6—C7	0.9 (4)	C2—C3—C12—O4	7.3 (3)
C4—C5—C6—Cl1	−178.9 (2)	C4—C3—C12—O4	−171.8 (2)
C5—C6—C7—C8	0.1 (5)	C12—O4—C13—C14	176.2 (4)
Cl1—C6—C7—C8	179.9 (2)		

### Hydrogen-bond geometry (Å, º)

**Table:**

*D*—H···*A*	*D*—H	H···*A*	*D*···*A*	*D*—H···*A*
C5—H5*A*···O3	0.93	2.24	2.872 (4)	125
